# Neuroimmunologie ohne Grenzen

**DOI:** 10.1007/s00115-024-01700-x

**Published:** 2024-09-26

**Authors:** Sven G. Meuth

**Affiliations:** Klinik für Neurologie, Universitätsklinikum Düsseldorf, Moorenstr. 5, 40225 Düsseldorf, Deutschland

Unser Verständnis der neuroimmunologischen Grundlagen einer Vielzahl von Erkrankungen des Nervensystems ist in den letzten Jahren sprunghaft gewachsen. Dies ist sowohl auf neueste technologische Entwicklungen in der Grundlagenwissenschaft als auch auf die zunehmende interdisziplinäre Zusammenarbeit in den Bereichen klinische Neurologie, Neurobiologie und Immunologie zurückzuführen. Die in diesem Sinne interdisziplinäre und translationale Erforschung der wechselseitigen Interaktion von Immun- und Nervensystem stellt derzeit einen der aufregendsten und am schnellsten wachsenden Bereiche immun- und neurobiologischer Forschung dar. Die Erkenntnisse der Neuroimmunologie der letzten Jahre haben doch gezeigt, dass es einen solchen stetigen Austausch zwischen unserem Nervensystem und unserem Immunsystem weit über die klassischen neurologischen Autoimmunerkrankungen hinaus gibt. Die aktuelle Ausgabe von *Der Nervenarzt* beschäftigt sich, wie auch der kommende Kongress der Deutschen Gesellschaft für Neurologie, schwerpunktmäßig mit dieser Entwicklung.

Es werden die wichtigsten Elemente sowohl des angeborenen als auch des adaptiven Immunsystems kurz vorgestellt, um dann an einigen ausgewählten Beispielen, wie der Multiplen Sklerose, dem Schlaganfall bis hin zu neurodegenerativen Erkrankungen, wie der amyotrophen Lateralsklerose (ALS), der Alzheimer-Erkrankung, der Parkinson-Erkrankung oder auch dem Glioblastom, die bisher bekannten immunologischen Grundlagen dieser neurologischen Erkrankungen zu skizzieren.

Auch wenn der Schlaganfall sicherlich keine klassische neuroimmunologische Erkrankung darstellt, ist es interessant zu sehen, wie immer neue entzündliche Mechanismen in diesem Kontext beschrieben werden. Eine 2021 in *The Lancet* publizierte Studie [1] zeigte, dass Schlaganfälle global ursächlich sind für den mit Abstand größten Verlust an gesunden Lebensjahren, den sog. DALYs („disability-adjusted life years“), also den verlorenen Jahren durch vorzeitigen Tod oder durch ein Leben mit Beeinträchtigung. Dabei besteht durchaus Anlass zu einem gewissen Optimismus. So wurden mit der systemischen Thrombolyse und der Thrombektomie Akuttherapien etabliert, die zu einem verbesserten Outcome der betroffenen PatientInnen führen. Zugleich gibt es Fortschritte bei der Prophylaxe, was wiederum zu einer Abnahme der auf das Lebensalter bezogenen Häufigkeit des Schlaganfalles in den westlichen Industrienationen beigetragen hat [2]. Hierbei spielt die Modifikation vaskulärer Risikofaktoren wie der arteriellen Hypertonie, Bewegungsmangel, Rauchen und Diabetes mellitus eine bedeutsame Rolle. Neben diesen häufigen klassischen Ursachen von Schlaganfällen besteht jedoch eine Reihe seltenerer, zum Teil immunvermittelter entzündlicher Schlaganfallursachen. Diese sind wenig erforscht, häufig schwer zu diagnostizieren, und aufgrund ihrer relativen Seltenheit fehlen große Therapiestudien, sodass sie uns NeurologInnen im klinischen Alltag häufig vor Schwierigkeiten stellen. Zudem sind, im Vergleich zu durch klassische Risikofaktoren verursachte Schlaganfälle, häufiger jüngere Menschen betroffen. Daher fokussieren sich mittlerweile einige Arbeitsgruppen auf die wichtigsten immunvermittelt entzündlichen Ursachen ischämischer Schlaganfälle. Hierbei werden sowohl erregerbedingte Schlaganfallursachen, wie die bakterielle Meningitis und die Endokarditis, als auch autoimmune Schlaganfallursachen einschließlich der Riesenzellarteriitis und der primären ZNS(Zentralnervensystem)-Vaskulitis erörtert. Daraus ergeben sich bereits heute relevante Aspekte für den klinischen Alltag, insbesondere der Diagnostik und Therapie.

Immunvermittelt entzündliche Ursachen ischämischer Schlaganfälle im Fokus

Wesentliche neue Erkenntnisse konnten auch im Bereich der peripheren neuroimmunologischen Erkrankungen gewonnen werden, die jüngst sogar zur Zulassung neuer Therapien, die gänzlich neuen Prinzipien folgen, geführt haben. Diese Neuzulassungen und die damit verbundenen therapeutischen Hoffnungen führen zu neuen praxisrelevanten Diskussionen und Updates zur Diagnostik und Therapie von Myositiden, der Myasthenia gravis, der chronisch-inflammatorischen demyelinisierenden Polyneuropathie (CIDP) sowie vaskulitischen Erkrankungen von Nerv und Muskel. Darüber hinaus bleiben Fragen zu spezifischen klinischen Herausforderungen wie neueste Therapieansätze, die Overlap-Myositiden, die seronegative Myasthenie und insbesondere auch der Stellenwert und Zusatznutzen der Nerven- und Muskelbiopsie Gegenstand der Diskussion. Die klinische Perspektive wird hierbei durch Einsichten erfahrener NeuropathologInnen bereichert, um ein besseres Verständnis der Pathophysiologie und daraus resultierende praxisrelevante Erkenntnisse zu ermöglichen.

Auch die Epilepsie ist keine klassische neuroimmunologische Erkrankung und dennoch spielen entzündliche Mechanismen auch in diesem Erkrankungsspektrum eine relevante Rolle. Nach Angaben der Weltgesundheitsorganisation (WHO) ist die Epilepsie eine der häufigsten schwerwiegenden neurologischen Erkrankungen im Hinblick auf Behinderung, Mortalität und verkürzter Lebensdauer [3]. Derzeit leiden weltweit mehr als 50 Mio. Menschen an Epilepsie, und etwa ein Drittel der Patienten – meist mit fokaler Epilepsie – hat eine unzureichende Anfallskontrolle mit anfallssupprimierenden Medikamenten (ASM; pharmakoresistente Epilepsie). Zu den häufigsten Ursachen einer durch Epilepsiechirurgie behandelten medikamentenresistenten fokalen Epilepsie gehören angeborene statische Fehlbildungen der kortikalen Entwicklung (MCD, einschl. fokaler kortikaler Dysplasie Typ I–III), erworbene niedriggradige epilepsieassoziierte Tumoren (LEAT) und die Hippokampussklerose (HS). Eine Verschiebung des exzitatorisch-inhibitorischen (EI) Gleichgewichts hin zur kortikalen Übererregbarkeit (HE) ist das grundlegende Kennzeichen der Epilepsie. Die zugrunde liegenden Mechanismen und Folgen von HE bei Epilepsie sind weiter unvollständig verstanden. Während die neuronalen Mechanismen der epileptischen HE eingehend untersucht wurden, deuten neuere Erkenntnisse darauf hin, dass extraneuronale, hauptsächlich immuninflammatorische und vaskuläre Mechanismen eine wichtige Rolle bei der Entwicklung und dem Fortschreiten der HE bei Epilepsie und ihren kognitiven und verhaltensbezogenen Begleiterkrankungen spielen. Damit zeigt sich, dass neuroimmunologische Mechanismen auch im Kontext der HE eine relevante Rolle spielen.

## Fazit

Die Forschung in der Neuroimmunologie ist dynamisch und expandiert kontinuierlich und führt damit zum einen zu einer Identifizierung neuer Krankheitsentitäten und zum anderen zu einem tieferen Verständnis der neuroimmunen Wechselwirkungen. Dieses Wissen wiederum führt zu spezifischeren und wirksameren therapeutischen Ansätzen, die das Gleichgewicht zwischen Immunüberwachung und der Vermeidung dysregulierter Immunität berücksichtigen. Während die Charakterisierung von Erkrankungen wie der Multiplen Sklerose, der Myasthenia gravis oder der Neuromyelitis-optica-Spektrumerkrankung (NMOSD) und das breitere Verständnis der Immunantworten neue therapeutische Möglichkeiten eröffnen, bleiben viele Fragen offen, die nur durch weitere grundlagenwissenschaftliche Forschung, geeignete experimentelle Modelle und translationale Strategien beantwortet werden können. Diese wissenschaftliche Programmatik könnte allerdings auch dazu beitragen, neuroimmunologische Mechanismen bei primär nichtentzündlichen neurologischen Krankheitsbildern zu beschreiben, was wir am Beispiel des Schlaganfalls und der Epilepsie in dieser Ausgabe diskutieren werden.

## Interessenkonflikt

Sven Meuth erhält Honorare für Vorträge sowie Reisekosten für die Teilnahme an Sitzungen von Academy 2, Alexion, Argenx, Almirall, Amicus Therapeutics Germany, Bayer Health Care, Biogen, BioNtech, BMS, Celgene, Datamed, Demecan, Desitin, Diamed, Diaplan, DIU Dresden, DPmed, Gen Medicine and Healthcare products, Genzyme, Hexal AG, IGES, Impulze GmbH, Janssen Cilag, KW Medipoint, MedDay Pharmaceuticals, Merck Serono, MICE, Mylan, Neuraxpharm, Neuropoint, Novartis, Novo Nordisk, ONO Pharma, Oxford PharmaGenesis, QuintilesIMS, Roche, Sanofi-Aventis, Springer Medizin Verlag, STADA, Chugai Pharma, Teva, UCB, Viatris, Wings for Life international und Xcenda. Seine Forschung wird gefördert vom Bundesministerium für Bildung und Forschung (BMBF), Bundesinstitut für Risikobewertung (BfR), der Deutschen Forschungsgemeinschaft (DFG), der Else-Kröner-Fresenius-Stiftung, dem Deutschen Akademischen Austauschdienst, dem Gemeinsamen Bundesausschuss (G-BA), der Hertie Stiftung, dem Interdisziplinären Zentrum für Klinische Forschung (IZKF) Münster, der Deutschen Stiftung Neurologie und Alexion, Almirall, Amicus Therapeutics Germany, Biogen, Diamed, DGM e.V., Fresenius Medical Care, Genzyme, Gesellschaft von Freunden und Förderern der Heinrich-Heine-Universität Düsseldorf e.V., HERZ Burgdorf, Merck Serono, Novartis, ONO Pharma, Roche, und Teva.
